# Aortic annular enlargement techniques using an original patch for prosthetic valve endocarditis

**DOI:** 10.1186/s12872-020-01518-w

**Published:** 2020-05-25

**Authors:** Kanji Matsuzaki, Yusuke Iguchi, Toru Tsukada, Akito Imai, Taisuke Konishi, Yasunori Watanabe

**Affiliations:** Department of Cardiovascular Surgery, Hitachi General Hospital, 2-1-1 Jonan, Hitachi, Ibaraki, 317-0077 Japan

**Keywords:** Prosthetic valve endocarditis, Periannular abscess, Aortic annular enlargement technique, Composite three-layer patch

## Abstract

**Background:**

Prosthetic valve endocarditis (PVE) is a serious complication, and it is difficult to treat marked adhesion and infectious tissue.

**Case presentation:**

There were four patients with aortic PVE, whose ages ranged from 59 to 80 years. In all patients, transoesophageal echocardiography revealed periannular abscess formation. We applied aortic annular enlargement techniques using a composite three-layer patch to repair the defects after radical debridement of the abscesses, and then replaced the prosthetic valves on the reconstructed annuli. All patients received antibiotics after surgery and recovered well without recurrence.

**Conclusions:**

The aortic annular enlargement techniques provided a good field of vision at the complicated annulus, and our original patch was useful for repairing the aortic annulus and its surrounding apparatus.

## Background

Prosthetic valve endocarditis (PVE) is a serious complication with an operative mortality ranging from 13 to 37% [1, 2]. In such surgeries, it is difficult to treat marked adhesion and infectious tissue. In response, we applied aortic annular enlargement techniques using a composite three-layer patch and replaced the prosthetic valves on the reconstructed annuli. To our knowledge, this is the first report of this aortic annular repair method for PVE.

## Case presentation

**Fig. 1:**
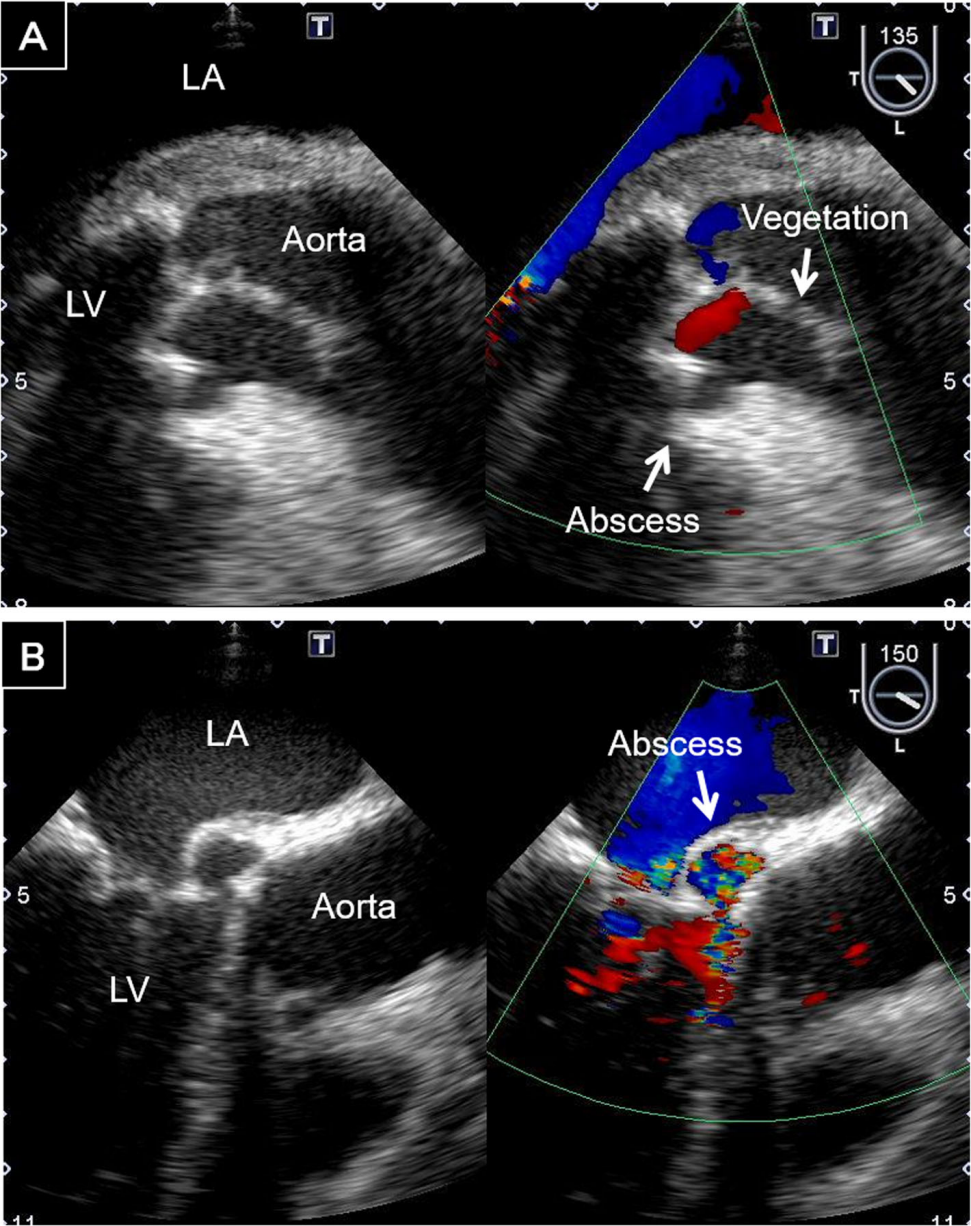
Transoesophageal echocardiogram. **a** A periannular abscess in the anterior annulus and vegetation growing from the aortic valve. **b** A periannular abscess in the posterior annulus. LA, left atrium; LV, left ventricle

There were four recent patients with aortic PVE, two men and two women, whose ages ranged from 59 to 80 years. One patient had methicillin-resistant *Staphylococcus epidermidis*, and the microorganisms in the others were methicillin-sensitive. In all patients, transoesophageal echocardiography revealed periannular abscess formation in the aortic annuli (Fig. 1a, b). Therefore, aortic annular enlargement techniques were applied to obtain a good field of vision at the aortic annulus. We performed redo aortic valve replacement with anterior or posterior repair of the aortic root on two patients each.

### Surgical technique

#### Anterior repair

**Fig. 2:**
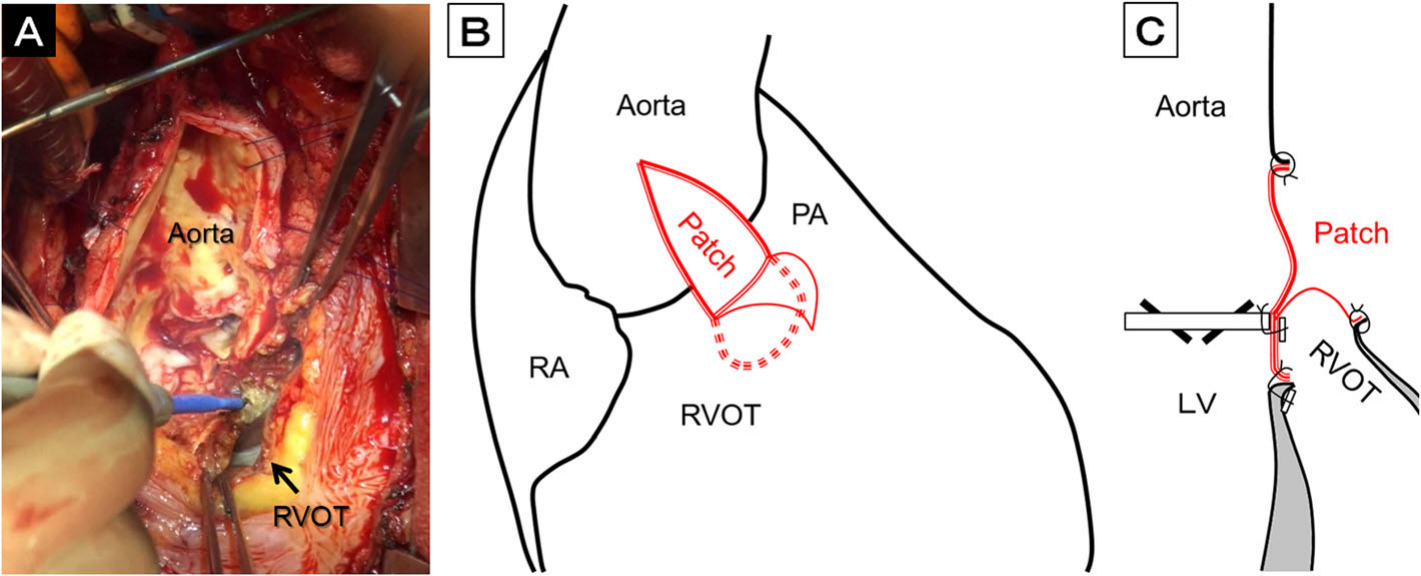
**a** Intraoperative view at the aortic annulus during anterior repair. **b, c** Schema of the anterior repair using a composite three-layer patch. LV, left ventricle; PA, pulmonary artery; RA, right atrium; RVOT, right ventricular outflow tract

An anterior annular enlargement technique was applied for the major abscess occupying the anterior side of the aortic annulus between the right and left coronary ostia. Following median re-sternotomy, cardiopulmonary bypass was established with arterial perfusion via the femoral artery and bicaval drainage. Dissection around the aortic root was poor due to marked adhesion. The left ventricle was vented by cannulation through the right superior pulmonary vein. Epicardial echocardiography was used to detect the exact sites of the right coronary artery ostium and the pulmonary valve. We performed anterior longitudinal aortotomy between them towards the aortic annulus, after clamping the ascending aorta under ventricular fibrillation (Video 1). Myocardial protection was achieved by initial selective cardioplegia, followed by intermittent retrograde cardioplegia in 30 min. After vena cava snaring, the right ventricular outflow tract adhering to the aortic root was incised in order to obtain a good field of vision, and the infected valve was removed using a surgical scalpel and scissors. Radical debridement of the abscess was carried out as much as possible, which slightly dissected the interventricular septum (Fig. 2a).

A composite three-layer patch made by sandwiching a Dacron patch between two sheets of bovine pericardium was used to repair the aortic annulus. Three pieces of the sheets had been soaked in antibiotic solution, such as rifampicin, and bonded with fibrin glue. The composite patch was trimmed into the shape of a teardrop, and its bottom half was sutured to the interventricular septum by stretching several mattress sutures using 4–0 polypropylene with a pledget. Regarding minor abscesses on the opposite side, the cavities were filled with fibrin glue containing antibiotics and repaired with autologous pericardium. A new prosthetic valve was then implanted into the reconstructed annulus. Both the Dacron patch and the inner bovine pericardium of the top half were used together to close the aortotomy by placing continuous sutures with 4–0 polypropylene. The outer bovine pericardium was used to close the right ventricular outflow tract by placing continuous sutures with 4–0 polypropylene (Fig. 2b, c).

#### Posterior repair

**Fig. 3:**
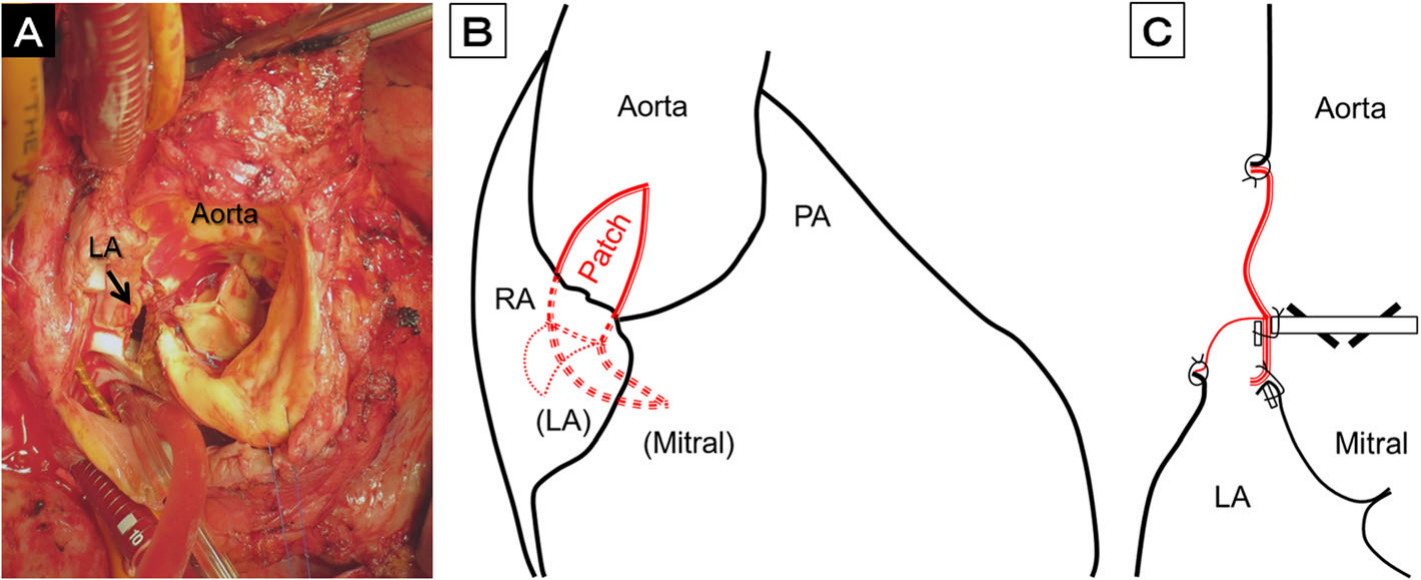
**a** Intraoperative view at the aortic annulus during posterior repair. **b, c** Schema of the posterior repair using a composite three-layer patch. LA, left atrium; PA, pulmonary artery; RA, right atrium

For major abscesses occupying the posterior side of the aortic annulus between the left and right coronary ostia, a posterior annular enlargement technique was used. Cardiopulmonary bypass and myocardial protection were performed in the same manner as in anterior repair. After vena cava snaring, we performed oblique aortotomy toward the major abscess (Video 2). The right atrium adhering to the aortic root was incised in order to obtain a good field of vision (Fig. 3a). The infected valve was removed and radical debridement of the abscess was performed as much as possible. As a result, the left atrium was opened along the aorto-mitral curtain.

A composite three-layer patch was trimmed appropriately, and its bottom half was sutured to the aorto-mitral curtain by stretching several mattress sutures using 4–0 polypropylene with a pledget. After repairing other minor abscesses as in anterior repair, a new prosthetic valve was implanted into the reconstructed annulus. Both the Dacron patch and inner bovine pericardium of the top half were used together to close the oblique aortotomy by placing continuous sutures with 4–0 polypropylene. The outer bovine pericardium was used to close the left atrial wall by placing continuous sutures with 4–0 polypropylene (Fig. 3b, c).

### Clinical result

One patient was provided with a permanent pacemaker because of atrioventricular block. All patients received antibiotics for 6 weeks after surgery and recovered well without recurrence. One patient underwent mitral valve replacement for deteriorating mitral regurgitation 5 years after the surgery, but died of advanced gastric cancer in the 6th year. The other three patients are alive without recurrence at 6 years or longer postoperatively.

## Discussion and conclusions

Radical debridement of the infected tissue and reconstruction of the damaged annulus are essential for PVE surgery. Complete debridement was reported to be more important than the materials used for annular repair and valve replacement [3]. When the whole aortic annulus is destroyed in PVE, aortic root replacement using a homograft or aortic valve conduit should be performed. However, most major abscesses do not expand over the entire annulus [4]. To such lesions, we apply patch repair of the aortic annulus followed by valve replacement using a standard tissue or mechanical prosthesis. Then, we use a composite patch soaked in antibiotic solution and fibrin glue containing antibiotics. The effectiveness of this type of procedure has been reported [3]. In our patients, endocarditis has not recurred for more than 5 years after the surgeries.

In our experience, aortic annular enlargement techniques play an important role in providing a good field of vision at the aortic annulus in secondary aortic valve surgery [5, 6]. We applied such techniques to PVE patients with periannular abscess formation because a good field of vision is essential for extensive debridement and reconstruction of the aortic annulus. Indeed, we used the techniques in 4 of 11 PVE surgeries performed during last 10 years. When a major abscess occupied the anterior side of the annulus, we performed anterior longitudinal aortotomy at the aortic root, incising the right ventricular outflow tract together [7]. When a major abscess occupied the posterior side, we performed oblique aortotomy, incising the right atrium together. The aortotomies were then extended to the periannular abscesses, the radical debridement of which dissected the interventricular septum or the aorto-mitral curtain.

However, these techniques have potential risks in hemodynamic and arrhythmic fields. In anterior repair, special attention must be paid in order not to interfere with the right coronary artery ostium and the pulmonary valve. Intraoperative epicardial echocardiography is a useful option for detecting the exact sites. Debridement of a periannular abscess in the interventricular septum leading to atrioventricular block is another concern. Indeed, one of our patients was treated by permanent pacemaker implantation for conduction block. In posterior repair, the left coronary artery and the mitral valve are at risk for dysfunction. One patient exhibited postoperative mitral regurgitation requiring valve surgery 5 years after the surgery.

To repair the major defects after debridement of the periannular abscesses, we used a composite three-layer patch and replaced the prosthetic valves on the reconstructed annuli. The three-layer structure was useful for serial reconstruction of aortic annuli and surrounding apparatuses. Furthermore, the bovine pericardium was sufficiently flexible to repair the endocardium of both ventricular outflow tracts and the atria. The combination of a Dacron patch and bovine pericardium was sufficiently strong to repair the aortic root, and was superior in hemostasis. Although bovine pericardium alone may be satisfactory regarding strength and hemostasis, there is no convincing evidence in the long-term period. On the other hand, Dacron is the material used for aortic grafts with long-term high durability. Considering the long-term durability of the aortic patch, we have adopted a composite patch that consists of a Dacron patch and bovine pericardium. This is the first report of this method of aortic annular repair using a three-layer patch for PVE.

In conclusion, aortic annular enlargement techniques provided a good field of vision at the aortic annulus complicated by infection and adhesion, and a composite three-layer patch was useful for repairing such an aortic annulus together with its surrounding apparatus.

## Supplementary information

**Additional file 1: Video 1.** Anterior repair.

**Additional file 2: Video 2.** Posterior repair.

## Data Availability

The datasets used are available from the corresponding author upon reasonable request.

## Abbreviation

PVE: Prosthetic valve endocarditis
